# A case of atrial fibrillation due to *Momordica charantia* (bitter melon)

**DOI:** 10.4103/0256-4947.59372

**Published:** 2010

**Authors:** Ismail Erden, Serkan Ordu, Emine C. Erden, Sabri O. Caglar

**Affiliations:** a From the Medicine Faculty, Department of Cardiology, Duzce University, Duzce, Turkey; b From the Department of Cardiology, Adatip Hospital, Adapazari, Turkey

**To the Editor:** Eighty per cent of the population in developing countries continues to use traditional medicine for primary medical problems. *Momordica charantia* (bitter melon), belonging to the family Cucurbitaceae, has been frequently used as a medicine. In Turkish folk medicine, mature fruits of *Momordica charantia* are used externally for wound healing and orally for the treatment of peptic ulcer.^1^ *Momordica charantia* has been credited with antidiabetic, antiseptic, antioxidant, anti-inflammatory, hypocholesterolemic, hypotensive, and immunostimulant properties.^2^

A 22 year-old man was admitted to our emergency department with complaints of palpitation and weakness. He had begun to use *Momordica charantia* two days before his admission for his dyspeptic complaints. He had crushed *Momordica charantia* and drunk two tablespoons of its juice three times a day, and had also drunk *Momordica charantia* juice on the morning of admission. The patient had no medical history of smoking, alcohol use, surgery, palpitation, coronary arterial disease, hypertension, diabetes, or chronic bronchitis. Physical examination revealed an arterial blood pressure of 100/70 mm Hg. The patient had a normal complete blood count, arterial blood gases, blood electrolyte (Na, K, Mg, Ca), and serial cardiac marker levels. Thyroxin and triiodothyronine concentrations and the findings of thyrotropin-releasing hormone tests were normal. Electrocardiography (ECG) showed atrial fibrillation (AF) with rapid ventricular response, approximately 136 beats/min (Figure 1a). Intravenous metoprolol was given to decrease the heart rate and intravenous amiodarone was administered for medical cardioversion

**Figure 1a:**
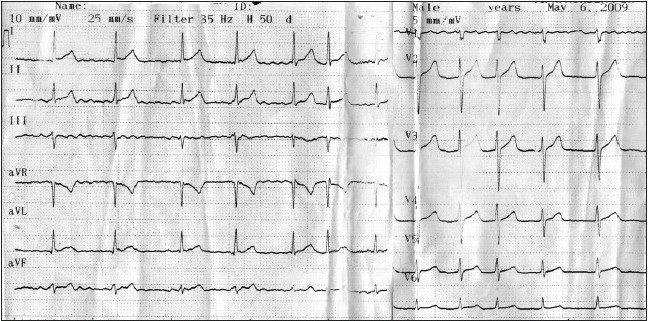
Electrocardiography showed atrial fibrillation with rapid ventricular response

We administered intravenous amiodarone for medical cardioversion and sinus rhythm was restored after ten hours of infusion (Figure 1b). Because of AF lasted few than 2 days, anticoagulation therapy wasn't given to the patient. During continuous ECG monitoring in the emergency department, no recurrence of arrhythmia was observed, and transthoracic echocardiography found no structural or functional anomaly. Finally, the use of *Momordica charantia* was the only etiology that could explain the episode of AF. Hence, termination of the use of MC was recommended and the patient was discharged without any medication. The Naranjo criteria classify the probability that an adverse event is related to drug therapy based on a list of weighted questions, which examine factors such as the temporal association of drug administration and event occurrence, alternative causes for the event, drug levels, dose – response relationships and previous patient experience with the medication.^3^ The Naranjo adverse drug reaction is assigned to a probability category from the total score as follows: *definite* if the overall score is 9 or greater, *probable* for a score of 5-8, possible for 1-4 and *doubtful* if the score is 0. The patient had six points according to the Naranjo adverse drug reaction scale, which indicated a probable casual association. At the three-month followup visit, ECG showed normal sinus rhythm, and the patient reported no cardiac symptoms during the time until the visit.

**Figure 1b:**
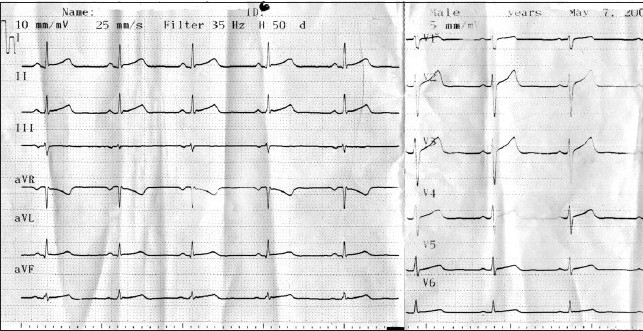
Electrocardiography after cardioversion: sinus rhythm.

*Momordica charantia* has been shown to ameliorate diet-induced obesity and insulin resistance, and has been reported to have beneficial effects such as lowered plasma lipid and blood sugar levels in animal studies.^2^ *Momordica charantia* contains biologically active chemicals that include glycosides, saponins, alkaloids, fixed oils, triterpenes, proteins, and steroids. Several phytochemicals such as momorcharins, cryptoxanthin, cucurbitins, cycloartenols, elaeostearic acids, erythrodiol, galacturonic acids, goyasaponins, and multiflorenol have also been isolated from this plant.^4^

*Momordica charantia* has been shown to be safe in humans at a dose of 20 mg/kg body weight.^5^ It has not been shown to be associated with nephrotoxicity, hepatotoxicity, or any adverse influence on food intake, growth organ weights, or hematological parameters. However, toxicity and even death have been reported in laboratory animals when high doses of the extracts were administered intravenously or intraperitoneally. The fruits and seeds have demonstrated greater toxicity than the leaves or aerial parts of the plant. Some documented adverse effects of *Momordica charantia* are hypoglycemic coma and convulsions in children, reduced fertility in mice, a favism-like syndrome, increases in gamma-glutamyltransferase and alkaline phosphatase levels in animals, and headaches.^2^

AF is the most common type of arrhythmia in adults. Cardiac conditions associated with the development of AF are hypertension, rheumatic mitral valve disease, coronary artery disease, and congestive heart failure. Noncardiac causes include hyperthyroidism, hypoxic pulmonary conditions, surgery, and alcohol intoxication.^6^ We carefully ruled out cardiac and noncardiac organic pathologies that could have caused paroxysmal AF in this patient. The patient's palpitations developed in the 48 hours before his admission and the patient had no history of AF. Sinus rhythm was restored in the patient after the administration of antiarrhythmic medication. ECG showed normal sinus rhythm after three months of follow-up. As no cardiovascular rhythm disorders have been reported due to ingestion of bitter melon seeds, our case is noteworthy because it draws attention to the possibility that MC use can cause paroxysmal AF.
